# Clarifying the Cryptic Host Specificity of *Blastocystis* spp. Isolates from *Alouatta palliata* and *A*. *pigra* Howler Monkeys

**DOI:** 10.1371/journal.pone.0169637

**Published:** 2017-01-05

**Authors:** Claudia Villanueva-Garcia, Elias Jose Gordillo-Chavez, Eduardo Lopez-Escamilla, Emilio Rendon-Franco, Claudia Irais Muñoz-Garcia, Lilia Gama, Williams Arony Martinez-Flores, Nayeli Gonzalez-Rodriguez, Mirza Romero-Valdovinos, Hilda Diaz-Lopez, Jose Galian, Guiehdani Villalobos, Pablo Maravilla, Fernando Martinez-Hernandez

**Affiliations:** 1 Departamento de Zoologia y Antropologia Fisica, Facultad de Veterinaria, Universidad de Murcia, Murcia, España; 2 Departamento de Ecologia del Paisaje y Cambio Global, Centro de Investigacion para la Conservacion y Aprovechamiento de Recursos Tropicales, Universidad Juarez Autonoma de Tabasco, Villahermosa, Tabasco, Mexico; 3 Hospital General “Dr. Manuel Gea Gonzalez”, Secretaria de Salud, Ciudad de Mexico, Mexico; 4 Departamento de Produccion Agricola y Animal, Universidad Autonoma Metropolitana, Unidad Xochimilco, Ciudad de Mexico, Mexico; 5 Escuela Nacional de Ciencias Biologicas, Instituto Politecnico Nacional, Ciudad de Mexico, Mexico; 6 Departamento de Ecologia Evolutiva, Instituto de Ecologia, Universidad Nacional Autonoma de Mexico, Ciudad de Mexico, Mexico; University of Illinois at Urbana-Champaign, UNITED STATES

## Abstract

Although the presence of cryptic host specificity has been documented in *Blastocystis*, differences in infection rates and high genetic polymorphism within and between populations of some subtypes (ST) have impeded the clarification of the generalist or specialist specificity of this parasite. We assessed the genetic variability and host specificity of *Blastocystis* spp. in wild howler monkeys from two rainforest areas in the southeastern region of Mexico. Fecal samples of 225 *Alouatta palliata* (59) and *A*. *pigra* (166) monkeys, belonging to 16 sylvatic sites, were analyzed for infection with *Blastocystis* ST using a region of the small subunit rDNA (SSUrDNA) gene as a marker. Phylogenetic and genetic diversity analyses were performed according to the geographic areas where the monkeys were found. *Blastocystis* ST2 was the most abundant (91.9%), followed by ST1 and ST8 with 4.6% and 3.5%, respectively; no association between *Blastocystis* ST and *Alouatta* species was observed. SSUrDNA sequences in GenBank from human and non-human primates (NHP) were used as ST references and included in population analyses. The haplotype network trees exhibited different distributions: ST1 showed a generalist profile since several haplotypes from different animals were homogeneously distributed with few mutational changes. For ST2, a major dispersion center grouped the Mexican samples, and high mutational differences were observed between NHP. Furthermore, nucleotide and haplotype diversity values, as well as migration and genetic differentiation indexes, showed contrasting values for ST1 and ST2. These data suggest that ST1 populations are only minimally differentiated, while ST2 populations in humans are highly differentiated from those of NHP. The host generalist and specialist specificities exhibited by ST1 and ST2 *Blastocystis* populations indicate distinct adaptation processes. Because ST1 exhibits a generalist profile, this haplotype can be considered a metapopulation; in contrast, ST2 exists as a set of local populations with preferences for either humans or NHP.

## Introduction

*Blastocystis* spp. is a common parasite that colonizes the intestines of many animals, including mammals, reptiles and birds. Although this microorganism is the most common eukaryotic parasite identified in human feces, its pathogenic role remains controversial. While some studies have reported that this parasite is associated with the development of cutaneous and intestinal disorders, other studies report no clinical manifestations [1–5]. In addition, *Blastocystis* exhibits high genetic polymorphism, presenting at least 17 subtypes (ST). The ST1-ST4 are frequently observed in humans but can also occur in birds, pigs, cows, dogs, rats and non-human primates (NHP). ST5 is common in pigs, and ST6 and ST7 are common in birds, while ST8 is documented in NHP and ST9 is only detected in humans. The ST10-ST17 have never been identified in people [2, 6, 7]; although, a recent study showed that ST12 may occur in humans [8]. The potential zoonotic transmission of *Blastocystis* has been under debate, as studies have reported dissimilar results [9–15]. In Kathmandu, Nepal, the potential transmission of *Blastocystis* between local rhesus monkeys (*Macaca mulatta*) and children was assessed; the authors detected three subgroups of ST2 shared between the children and the monkeys, suggesting that the local rhesus monkeys might serve as a potential source for human ST2 infections [12]. Studies primarily concentrating on the genetic variability of *Blastocystis* recovered from humans and several primate genera, including Old and New World monkeys and prosimians, have confirmed the cryptic host specificity of ST1 and ST3. These parasites exhibit only minimal overlap between the sources, indicating that some ST3 isolates have adapted to NHP, while others have adapted to humans. Furthermore, reflecting considerable overlap in ST2, haplotypes exists among humans and NHP, and transmission among these species may occur in zoological parks and animal sanctuaries [11, 14, 16]. In contrast, a study performed in the Rubondo Island National Park, Tanzania, where different autochthonous and introduced NHP live together, ST1-ST3 and ST5 were detected. Interestingly, the chimpanzees (*Pan troglodytes*) were parasitized only by ST1, which formed a unique phylogenetic clade. This finding suggested that Rubondo chimpanzees were colonized by a single, host-specific *Blastocystis* strain that circulates among the members of the group and that transmission of this genotype does not occur between primate populations and thus does not constitute a reservoir for human infections [13]. Recently, Helenbrook et al. [15] studied fecal samples from humans and howler monkeys (*Alouatta palliata aequatorialis*) from Ecuador living in allopatric areas at close proximity. *Blastocystis* ST1-ST3 were detected in the human samples, while all monkeys had ST8, thus questioning the zoonotic potential of ST8. Furthermore, howlers are particularly interesting because these animals appear to be more sensitive to infectious diseases, such as yellow fever and gastrointestinal parasites [17–19]. Different howler species live in forest fragments, disturbed habitats, and in close proximity to human populations [17, 19].

For many pathogens, the presence of multiple host species has important epidemiological and evolutionary implications, i.e., alternative host species might be reservoirs of a disease extinct in one host. In addition, parasites that interact with multiple host species may be less locally adapted, and consequently, these organisms are expected to be less specific. In addition, the assessment of host ranges can be hindered by the presence of cryptic species: even if a parasite is able to infect different host species, differences in infection rates among the alternative hosts might be interpreted as a consequence of a local adaptation process, leading to the preference for certain host species over others [20]. Hence, the identification of a generalist or specialist profile of a parasite for its host is relevant for understanding its prevalence, transmission and other biological features. Therefore, the aim of the present study was to assess the genetic variability and host specificity of *Blastocystis* spp. populations in howler monkey species *A*. *palliata* and *A*. *pigra* from two sylvatic areas of Mexico.

## Materials and Methods

### Study population

The genus *Alouatta* comprises 6 species widely distributed between Mexico and Argentina in allopatric or sympatric patterns. *A*. *palliata* and *A*. *pigra* howler monkeys are important inhabitants of some tropical rainforest areas in the southeastern region from Mexico, and both species are critically endangered and primarily distributed in states of Chiapas, Campeche, Quintana Roo, Tabasco, Veracruz and Yucatan. These arboreal monkeys live in troops, and their population densities vary considerably, ranging from <10 to approximately 100 individuals per km^2^ [17]. Although these monkeys are largely folivorous, their indiscriminate diet is likely one of the main reasons that these animals are adaptable to changing ecological landscapes, whether natural or anthropogenic [19].

### Sample strategy and study area

The present study was conducted in Tabasco and Chiapas states in the southeastern region of Mexico. Tabasco is a large flat coastal alluvial plain characterized by poor drainage and large areas of permanently or seasonally inundated terrains. The Grijalva and lower Usumacinta Rivers water the eastern and central parts of the plain. The weather is warm and moist, with a high constant temperature of ~26°C and an annual range of rainfall of 2000 to 4000 mm H_2_O. Chiapas, the neighboring state, has areas with different climates, abundant rainfall and diverse topology. Furthermore, the study region is a mosaic formed by native vegetation patches and extensive urban areas, grasslands, crops, shrubs, flood areas, and riparian forests. The native vegetation comprises patches of evergreen forest and riparian forest, mangle and marsh areas [21]. Field collection occurred in 2014 and 2015. The sampling sites were selected among vegetation areas used by *Alouatta* species for feeding, and there is information available on the general landscape, hydrography and flood vulnerability recorded from Tabasco's Ecological Planning Program [22]. An arc-tool called “neighbor joining” in the Arcview 10.0 program was used to discriminate overlapping areas. Initially, we established a random minimum separation of 5 km between every population to avoid resampling the same population. This distance was established after considering the mobility reported for a group of *A*. *palliata*, which traveled less than 50 m on 12 of the 34 days during that these animals were followed. The *A*. *pigra* group traveled the same distance but on 15 of the 34 days [23]. After interviews with local people, subdivisions and fieldwork, the presence of these monkeys was corroborated when howler troops were visually located using binoculars or based on their vocalizations. Sampling sites were selected considering 16 buffer areas, ranging from 7,852 m as minimum to 13,230 m as maximum, with distances of 10 to 357 km between each buffer. These sites included 14 sites in Tabasco and two sites in Chiapas (Table 1). The sites where *A*. *palliata* and *A*. *pigra* populations showed allopatric distribution were separated by the Grijalva River, living on the west and east sides of the river, respectively, except for one site in Tabasco (site 3, corresponding to the Tapijulapa, Cascadas de Villa Luz and Kolenchen localities) where both monkey species live in a sympatric area. The municipality Emiliano Zapata in Tabasco (site 8a) was of particular interest because, at this site, there is a group of small islands in the middle of the Chaschoc-Seja Lagoon (with some howlers living on two of the islands), with only boat access from the Bertollini Ranch (site 8b). Although some areas showed different grades of anthropogenic disturbance, among the 16 sites, 9 sites are considered as conserved zones, including forest and wetlands, while the other sites are meadows without human interactions, except for site 10 (Parque La Venta/ Tabasco), in which there is a proximity to humans and their homes.

**Table 1:** Infection rates of *Blastocystis* subtype (ST) for howler monkey populations.

Site ID	Localities/State	Species	N^1^	% *Blastocystis* infection^2^	% *Blastocystis* ST^3^			Accession number of GenBank
					ST1	ST2	ST8	
1	Playon de la Gloria/CHIAPAS	*A*. *pigra*	9	11.1 (1/9)	-	100 (1/1)	-	KT591833
2	Reforma Agraria/CHIAPAS	*A*. *pigra*	40	60 (24/40)	-	100 (24/24)	-	KT591789-94, KT591804-05, KT591814-17, KT591799-803, KT591806-09, KT591811-13
3	Tapijulapa, Cascadas de Villa Luz, Kolenchen/ TABASCO	*A*. *pigra*	5	20 (1/5)	-	100 (1/1)	-	KT591831, KT591839, KT591769
		*A*. *palliata*	8	37.5 (3/8)		100 (3/3)		
4	Poana, Xicotencatl/ TABASCO	*A*. *pigra*	22	18.2 (4/22)	-	50 (2/4)	50 (2/4)	KT591786-87, KT591852-53
5	Pochitocal/ TABASCO	*A*. *pigra*	5	0	-	-	-	-
6	Cascadas de Agua Blanca/ TABASCO	*A*. *pigra*	3	66.6 (2/3)	50 (1/2)	50 (1/2)	-	KT591851
7	Rancheria Josefa Ortiz de Dominguez/ TABASCO	*A*. *pigra*	5	80 (4/5)	-	100 (4/4)	-	KT591795-98
8a	Islands, Xeha Lagoon, Emiliano Zapata/ TABASCO	*A*. *pigra*	34	47(16/34)	6.3 (1/16)	93.7 (15/16)	-	KT591850, KT591770-75, KT591777-85
8b	Bertollini Ranch, Emiliano Zapata/ TABASCO	*A*. *pigra*	16	37.5 (6/16)	16.6 (1/6)	83.3 (5/6)	-	KT591848, KT591820-23,KT591838
9	Los Pajaros/ TABASCO	*A*. *pigra*	4	25 (1/4)	-	100 (1/1)	-	KT591788
10	Parque La Venta/ TABASCO	*A*. *palliata*	8	100 (8/8)	-	100 (8/8)	-	KT591840-47
11	Cocoa plantation and Ranch Cali/ TABASCO	*A*. *palliata*	3	33.3 (1/3)	-	100 (1/1)	-	KT591835
12	Carlos Greene/ TABASCO	*A*. *palliata*	30	26.6 (8/30)	12. 5 (1/8)	87.5 (7/8)	-	KT591849, KT591824-30
13	Palestina/ TABASCO	*A*. *palliata*	4	25 (1/4)	-	100 (1/1)	-	KT591768
14	Tabasquillo/ TABASCO	*A*. *palliata*	7	28.6 (2/7)	-	50 (1/2)	50 (1/2)	KT591837, KT591854
15	San Juanito and Tres Brazos/ TABASCO	*A*. *pigra*	11	18.2 (2/11)	-	100 (2/2)	-	KT591818-19
16	Nueva Alianza and La Victoria /TABASCO	*A*. *pigra*	11	27.3 (3/11)	-	100 (3/3)	-	KT591834, KT591836, KT591832
		*A*. *pigra*	166	38.5 (64/166)	4.6 (3/64)	92.1 (59/64)	3.1 (2/64)	
		*A*. *palliata*	59	38.9 (23/59)	4.3 (1/23)	91.3 (21/23)	4.3 (1/23)	
	**Total**		**225**	**38.7 (87/225)**	**4.6 (4/87)**^**4**^	**91.9 (80/87)**^**5**^	**3.5 (3/87)**^**6**^	

^1^N, number of analyzed howlers.

^2^% *Blastocystis* infection, obtained as all positive samples for *Blastocystis*x100/N.

^3^% *Blastocystis* ST, obtained as positive samples for each STx100/all positive samples for *Blastocystis* in a specific site.

^4^ST1 vs ST2+ST8 for *A*. *palliata* or *A*. *pigra*; *p* = 0.9471, 95% Confidence interval (95%IC) = 0.09–28.5.

^5^ST2 vs ST1+ST8 for *A*. *palliata* or *A*. *pigra*; *p* = 0.4981, 95%IC = 0.29–9.17.

^6^ST8 vs ST1+ST2 for *A*. *palliata* or *A*. *pigra*; *p* = 0.7840, 95%IC = 0.71–20.85.

### Species identification and fecal sample collection

Species identification was performed using morphological characteristics: *A*. *pigra* is larger, and their hair color is completely black. Additionally, testes are evident in male *A*. *pigra* infants, unlike *A*. *palliata* males, in which the testicles do not descend until sexual maturity. The fur of *A*. *palliata* is not uniform in color and is typically dense, with golden flecks in the underarm region and areas with no pigment on the hands, feet and tail [24]. Fecal samples were collected using a non-invasive technique: waiting until the monkeys defecated. Only one sample per animal was collected. To this end, at least two persons monitored the howler monkey troops: one person recovered the fecal sample and the other person followed the howlers to obtain their approximate age, sex and some identification features of each individual, thereby guaranteeing no resampling. Additionally, we verified the species identification using mitochondrial and nuclear markers [25] and determined whether each sample and its corresponding monkey matched. All samples were fresh and carefully collected using gloves to avoid contamination from the soil, arthropods and vegetal detritus; only the top of each sample was saved and stored in 70% ethanol for further molecular analysis.

### DNA extraction and PCR assays

DNA was extracted from approximately 100 mg of each fecal sample using the ZR Fecal DNA MiniPrep Kit (Zymo Research, Irvine, USA). The primers used for end-point PCR assays amplified an ~500 bp region of the small subunit rDNA (SSUrDNA) gene [26], [27]. PCR amplifications were performed in a final volume of 25 μL, containing 6.25 pmol of each primer, 1X PCR buffer (8 mM Tris-HCl, pH 8, and 20 mM KCl), 2.4 mM MgCl_2_, 0.5 mM dNTPs, 0.01 mg BSA, and 1 U Taq DNA Polymerase (Promega). Up to 50 ng of DNA (~2 μL) was used as a template to amplify the genomic sequences. The following amplification conditions were used: 94°C for 5 min, followed by 36 cycles at 94°C-30 s, 54°C-30 s and 72°C-30 s, with a final extension step at 72°C for 10 min. The amplicons were assessed using electrophoresis on 1.2% agarose gels, after which the bands were purified using the AxyPrep PCR Clean-up Kit (Axigen Biosciences, CA, USA) and sequenced on both strands by a commercial supplier. Chromatograms were evaluated with Mesquite software using the Chromaseq package [28–29], with phred and phrap algorithms for base calling, assigning quality values to each one and assembling contigs [30–32].

### Phylogenetic reconstruction and genetic variation analysis

All sequences were subjected to BLAST search in the GenBank database, and the sequences obtained in this work were accessed with the numbers KT591768-KT591854. Multiple alignments were performed using the CLUSTAL W [33] and Muscle [34] programs, with manual adjustment in MEGA 5.05 software [35]. The best-fit model of nucleotide substitution was determined using the Akaike Information Criterion in Modeltest software, version 3.7 [36] and the Hasegawa Kishino Yano model with gamma distribution and invariable sites. Phylogenetic reconstruction using Bayesian inference was performed using Mr. Bayes 3.1.2 [37]. The analysis was performed over 10 million generations, with sampling trees every 100 generations. Trees with scores lower than those at the stationary phase (burn-in) were discarded. The trees that reached the stationary phase were collected and used to build consensus trees. Other sequences were collected from GenBank and used as subtype references and for population genetics analysis (S1 Table).

A Median Joining Network analysis [38] was performed using NETWORK 4.611(fluxus-engineering.com) with default settings and assumptions. Genetic diversity analyses within and between populations were performed using DnaSPv4 [39], and indices, such as nucleotide diversity (π), haplotype polymorphism (θ), gene flow (*Nm*),genetic differentiation index (F_ST_) and Tajima’s D test were obtained. These indices have been previously applied for population genetics studies in *Blastocystis* [40, 41]. They denote the average proportion of nucleotide differences between all possible pairs of sequences in the sample (π); the proportion of nucleotide sites expected to be polymorphic in any suitable sample from this region of the genome (θ); the movement of organisms among subpopulations (*Nm*); and the differentiation between or among populations (F_ST_). To support the data interpretation, populations strongly differentiated have an *Nm* < 1, whereas those with an *Nm* > 4 behave as a single panmictic unit. For F_ST_, the following commonly used values for genetic differentiation were considered: 0 to 0.049, small; 0.05 to 0.149, moderate; 0.150 to 0.25, great; and values above 0.26 indicate enormous genetic differentiation. Negative values for Tajima’s D test suggest a recent expansion process or an effect of purifying selection [42].

### Statistical analysis

Descriptive statistics are expressed as the mean and standard deviation (SD). Analysis using Student's t test for unrelated samples and Mantel–Haenszel test were applied, and 95% confidence intervals (95%CI) were also obtained. Data analysis was performed using SPSS software, version 15.0 (SPSS Institute, Chicago, IL).

### Ethics statement

All fecal samples were collected using a non-invasive technique after the monkeys defecated. Sampling and procedures were performed in accordance with the provisions of the Regulations of the Environment and Natural Resources Ministry and the Under-Secretary of Management for Environmental Protection NOM-059-SEMARNAT-2010; reference SGPA/DGVS/04725/13. Academic authorities of the Universidad Juarez Autonoma de Tabasco authorized the present study (reference number UJAT-2013-IB-43).

## Results

### Frequency of *Blastocystis* spp. in *A*. *palliata* and *A*. *pigra*

**Fig 1:**
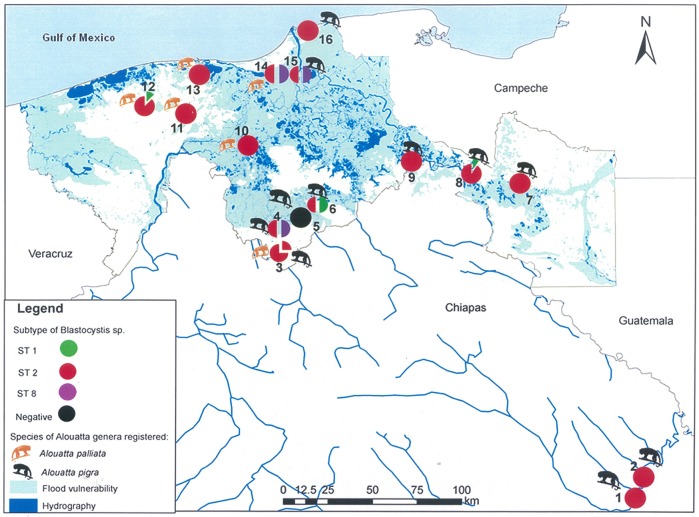
Field sampling sites, hydrography and frequency of *Blastocystis* ST in *Alouatta palliata* and *A*. *pigra* from Tabasco and Chiapas. The pie charts indicate those localities with positive samples and each colour represents a different ST; proportions of colours are according to ST frequencies.

In 14 sites from Tabasco and two sites from Chiapas, fecal samples from 225 howler monkeys, *A*. *palliata* (59) and *A*. *pigra* (166) were collected and analyzed. Table 1 summarizes the positive *Blastocystis* ST results for the animals from each site; only at site 5 (Pochitocal, Tabasco) were the monkeys not parasitized. The frequency of *Blastocystis* spp. was 38.7% (n = 87), 39% for *A*. *palliata* and 38% for *A*. *pigra;* ST2 was the most abundant (91.9%), followed by ST1 and ST8 with 4.6% and 3.5%, respectively. No association between *Blastocystis* ST and *Alouatta* species was observed. In addition, we identified infected howlers with similar frequencies and ST distributions in flooded and non-flooded areas (Fig 1).

### Phylogenetic reconstruction and genetic variation analysis

**Fig 2:**
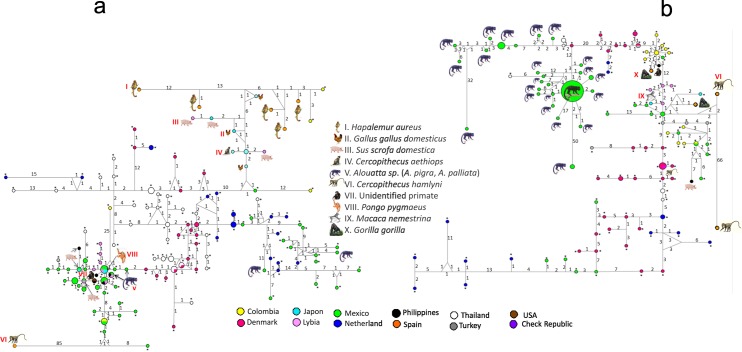
Haplotype networks for *Blastocystis*. Haplotype network trees using SSUrDNA sequences from different countries and hosts for ST1 (a) and ST2 (b). Numbers in branches refer to mutational changes; sizes of circles and colors are proportional to haplotype frequencies. For those animal haplotypes, an image and Roman reference numbers were included, while for human haplotypes, asterisks were added.

The contigs were assembled using both forward and reverse chromatograms from each sample, and analysis using Mesquite/Chromaseq software showed well-defined peaks without significant background noise. A Bayesian phylogenetic tree was built for SSUrDNA using all worldwide available sequences recorded in GenBank (S1 Fig). The sequences were grouped into the ST1, 2, and 8 clusters. The haplotype networks for ST1 and ST2 showed contrasting distributions: for ST1 (Fig 2A), several haplotypes from different mammals (humans, NHP, and pigs) and birds are homogeneously distributed in different countries, and in general, few mutational differences are present among them. Conversely, for ST2 (Fig 2B), a principal dispersion center grouped most of the Mexican howlers together, with the remaining sequences also closely distributed. Interestingly, some haplotypes showed high mutational differences, particularly in the NHP vs. human haplotypes. For example, for the Mexican sequences, three howler haplotypes diverged from the principal dispersion center by over 40 mutational changes, with the most divergent being a sample from one of the islands (site 8a). A similar phenomenon was observed for the ST2 sequences previously reported for *Cercopithecus hamlyni* from Spain [26], with one haplotype close to the human haplotypes, and another haplotype markedly distant, with up to 76 mutational changes.

**Fig 3:**
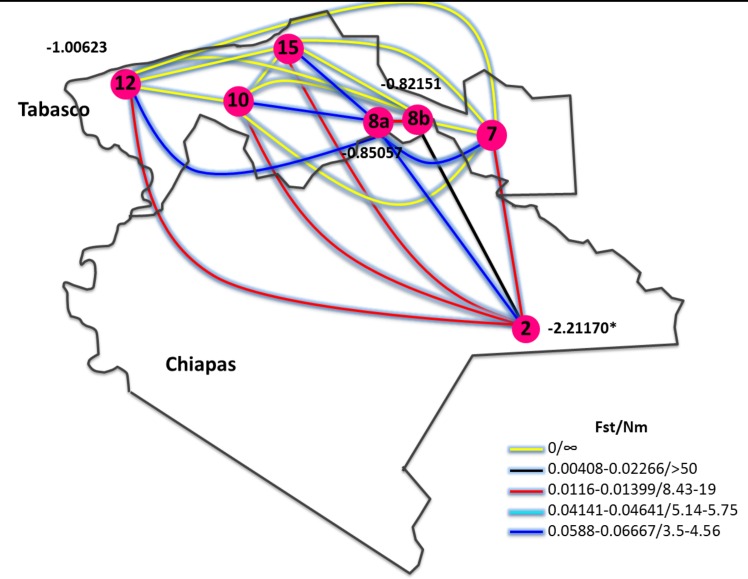
Schematic representation of interactions among population indexes. The gene flow (Nm), genetic differentiation index (F_ST_), and Tajima’s D values of *Blastocystis* ST by SSUrDNA analysis, according to different sampling sites; only those sites in which there were enough infected howlers to obtain the indexes are shown. The number together the sampling size circle, mean the Tajima’s D value. * *p*<0.01

Fig 3 shows the ranges of F_ST_ and *Nm* among the different sampling sites in Tabasco and Chiapas. Despite the great geographic distances between some of the populations, minimal differentiation and high gene flow were observed. As expected, the population with the lowest differentiation and gene flow was in the islands (site 8a), in contrast with site 8b, which showed F_ST_ and *Nm* values similar to those of the other sampling sites. The populations at sites 2, 8a, 8b and 12 yielded negative Tajima’s D values, but only for site 2, and this result was statistically significant (*p*<0.01).

Table 2 shows the comparisons between the indexes obtained for ST1 and ST2 of humans and NHP. The ST8 haplotype was not analyzed because of an insufficient number of sequences. These indexes support the topologies of the contrasting haplotype networks (Fig 2A and 2B). In the intra-ST comparison between humans and NHP, human *Blastocystis* ST1 populations had lower π values than those of NHP, whereas for ST2, human and NHP parasites had similar variability; despite the disparity between the number of available sequences in the GenBank of humans and NHP for ST1, the genetic diversity values were statistically significant. The *Nm* and F_ST_ values for ST1 and ST2 were inverted, suggesting that *Blastocystis* ST2 populations from humans and NHP are strongly differentiated with occasional gene flow, while scarcely any differentiation between ST1 parasite populations from humans and NHP was detected.

**Table 2:** Values of nucleotide diversity (π), haplotype polymorphism (θ), gene flow (*Nm*) and genetic differentiation index (F_ST_) for sequences of humans and HNP populations of *Blastocystis* ST1 and ST2.

	N	π±SD	p*	θ±SD	p*	F_ST_	*Nm*
ST1							
Humans	147	0.0241±0.0030	0.0001	0.0298±0.0080	0.0001	0.071	3.25
NHP	14	0.061±0.0169		0.0694±0.00026			
ST2							
Humans	94	0.0211±0.0022	0.247	0.0238±0.0070	0.0001	0.644	0.14
NHP	83	0.0204±0.0059		0.0774±0.0204			

*Student’s t-test, for independent samples.

## Discussion

The few reports on the molecular identification of *Blastocystis* ST in *Alouatta* monkeys [14, 15, 43] have shown dissimilar results for this NHP genus. The results obtained in the present study not only strengthen the existence of cryptic host specificity for ST1 and ST2 but also show that these ST have different population structures, with certain haplotypes of ST2 showing a preference for either humans or NHP. Generalist parasites infecting several host species can actually be cryptic parasite species, each characterized by a different degree of host specificity, particularly influenced by local adaptations that have led to a preference for certain host species over others and have zoonotic implications. Therefore, the generalist profile of some parasites may be a key process for the maintenance of their genetic diversity and population diversification [16, 20, 44–46].

In the present study, we observed that ST1 exhibits a generalist profile because there was minimal differentiation between parasite populations from humans and NHP, as well as substantial gene flow between them, suggesting that ST1 landscapes resemble a metapopulation (ensemble of interacting populations with a finite lifetime) [47–50]. In contrast, some *Blastocystis* ST2 haplotypes have diverged similar to a set of local populations with preferences towards certain host species (humans or NHP). Both population structures have relevance for understanding the prevalence and transmission of *Blastocystis* ST. According to these findings, the putative metapopulation structure of ST1 implies a link with the processes of population turnover, extinction and establishment of new populations [48], i.e., *Blastocystis* ST1 may have the capacity to recolonize vacant niches, such as new hosts, even of different species, supporting the zoonotic transmission of this parasite [6–9, 11–14]. In addition, Sanchez-Aguillon et al. [27] documented reinfection by *Blastocystis* ST1 in an asymptomatic patient who received anti-parasitic treatment and follow-up three months earlier.

However, the existence of ST2 as a set of resident populations locally adapted by each parasite population would support the preference for infecting either humans or NHP. Such a process of local adaptation is supported by Helenbrook et al. [51] who observed that *Blastocystis* was the unicellular parasite most frequent (60%) in samples of *A*. *palliata aequatorialis* howlers from Ecuador. Subsequently, in another study performed in similar populations of these howler monkeys, 68% of these animals were infected with *Blastocystis* ST8, while humans living in close proximity were infected with ST1, ST2 and ST3 [15]. Interestingly, although these subtypes (ST1-ST3) are distributed worldwide, they are common in America [8, 40, 41]. In the present study, 39% and 38% of *A*. *palliata* and *A*. *pigra* howlers, respectively, were primarily infected with *Blastocystis* spp., and of these, 91.3% of *A*. *palliata* and 92.1% of *A*. *pigra* howler monkeys were harboring *Blastocystis* ST2. Other relevant findings regarding the host specificity of ST2 were derived from the phylogenetic tree of parasites from Rubondo Island, Tanzania [13], in which the cluster for ST2 showed two clades for *Blastocystis*, those from humans and those from NHP.

In addition, as cited above, local adaptations are relevant for the maintenance of genetic diversity and population diversification. The high degree of intra-ST genetic polymorphism has been reliably documented, particularly for ST1 and ST3 [12, 14, 16, 41, 43–44], suggesting that populations of recent origin have undergone a radiation process [52]. However, the great divergence of some haplotypes for ST1 and ST2 observed in the present study deserves special attention. The presence of divergent haplotypes with more than 40 mutational differences between sequences from NHP of Spain and Mexico, particularly in a monkey from an island (site 8a), suggests the island-continent model described by Wright [53], in which many finite subpopulations (equivalent to the continent) are present, including the source of migrants to the island. When the amount of gene flow and the population size on the islands are both large, the allele frequency on the islands will soon become similar to that of the continent. However, if the population size on the island is small or if the rate of gene flow is low (as in the present study), then genetic drift could lead to random changes in allele frequency. As a result, the allele frequency on the islands may differ significantly from that on the continent and that of the migrants [54]. Alternatively, the population size could correspond to an epidemic population structure such as that depicted by Maynard-Smith et al. [55], in which there is frequent recombination within all members of the population, such that the structure is a net rather than a tree.

It has been observed that the use of more than one genetic marker has facilitated the clarification of the differences between organism populations. However, some studies of genetic variability focused only on the SSUrDNA gene analysis have shown that this marker is sensitive enough to resolve phylogenetic relationships, population differentiation events and cryptic infections in both *Blastocystis* and other parasites [8, 9, 14, 36, 56–58].

Since mixed ST infections are common [43, 59–60], we used Mesquite/Chromaseq software to identify co-infections in the howler monkeys as this technique has been used to reduce bias and misinterpretations during sequence analysis [28–32]; in this way, this analysis could distinguish a double profile in the chromatogram sequences, suggesting mixed infections based on different ST. However, future use of ST-specific primers, cloning, and SSCP analysis should be performed to confirm the presence or absence of ST co-infections.

Although a definitive mode of transmission for *Blastocystis* has not been identified, it has been suggested that transmission occurs orally through water, food or direct contact with the parasite; here, *Blastocystis* transmission apparently occurs through water [61], but wild howler monkeys have rarely been observed drinking river water. Instead, these animals use arboreal water cisterns [62], suggesting that transmission may occur when monkeys are in contact with contaminated leaves or other non-arboreal elements (i.e., lianas, vines and epiphytes) [63–64], wet ground or sewage during their movement between trees. Nevertheless, transmission may occur when monkeys drink water directly from the ground; Serio-Silva and Rico-Gray observed this behavior for *A*. *palliata* and *A*. *pigra* feeding and traveling on the ground, in small patches in locations with scarce trees, and during the dry and wet seasons in Balancan, Tabasco [65].

The results of the present study demonstrate that howler monkey populations of *A*. *palliata* and *A*. *pigra* with locally adapted *Blastocystis* ST1 and ST2 populations exhibit distinct generalist and specialist types of host specificity. In addition, these data suggest that the *Blastocystis* ST2 populations in humans are highly differentiated from those of NHP, while the ST1 populations are only minimally differentiated. The host generalist and specialist specificities exhibited by ST1 and ST2 *Blastocystis* populations are thus distinct processes. Because ST1 exhibits a generalist profile, this haplotype can be considered a metapopulation; in contrast, *Blastocystis* ST2 exists as a set of local populations with preferences for either humans or NHP.

Furthermore, it is important to highlight the use of population genetics studies in the epidemiology of this eukaryote because these studies may clarity some aspects of host-parasite relations; for example, how genetic variability within a subtype is reflected in phenotypic and functional variability and its potential role on the symptoms of the parasite carriers [66].

## Supporting Information

S1 Fig Phylogenetic inference of *Blastocystis* spp. Bayesian phylogenetic tree using a fragment of SSUrDNA sequences; the values of the nodes indicate posterior probabilities values using 10 million generations. ST and GenBank accession numbers are shown, as well as identification of each sample.
(TIF)

S1 Table GenBank accession number of sequences used in genetics population analysis. (XLSX)
