# Supplementary figures and images for: Clarifying the Cryptic Host Specificity of Blastocystis spp. Isolates from Alouatta palliata and A. pigra Howler Monkeys
Source: PLoS One. 2017 Jan 5;12(1):e0169637. doi: 10.1371/journal.pone.0169637 (PMC5215913; doi:10.1371/journal.pone.0169637)

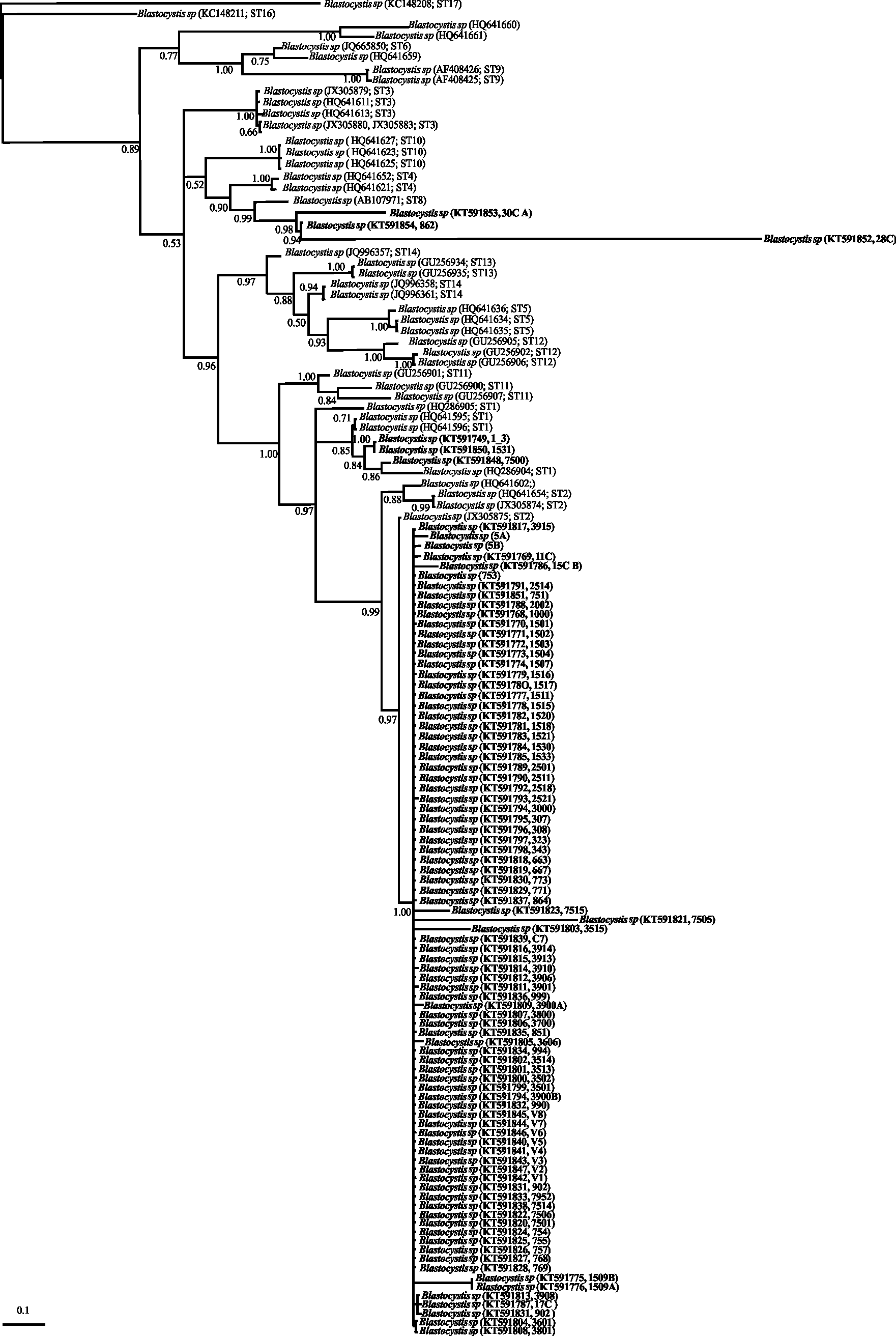

Supplement: S1 Fig — Bayesian phylogenetic tree using a fragment of SSUrDNA sequences; the values of the nodes indicate posterior probabilities values using 10 million generations. ST and GenBank accession numbers are shown, as well as identification of each sample. (TIF) [file pone.0169637.s001.tif]
